# Preparation of Lung-Targeting, Emodin-Loaded Polylactic Acid Microspheres and Their Properties

**DOI:** 10.3390/ijms15046241

**Published:** 2014-04-11

**Authors:** Xiaohong Chen, Zifeng Yang, Renshan Sun, Ziyao Mo, Guangyao Jin, Fenghuan Wei, Jianmin Hu, Wenda Guan, Nanshan Zhong

**Affiliations:** 1 State Key Laboratory of Respiratory Diseases, the First Affiliated Hospital of Guangzhou Medical University, Guangzhou Medical University, Guangzhou 510230, China; E-Mails: pharmacxh@yahoo.com (X.C.); jeffyah@hotmail.com (Z.Y.); moziyao@163.net (Z.M.); hispring112233@163.com (G.J.); hujming1972@163.com (J.H.); wenda@gird.cn (W.G.); 2 Department of Pharmacology, College of Pharmacy, Third Military Medical University, Chongqing 400038, China; 3 School of Traditional Chinese Medicine, Southern Medical University, Guangzhou 510515, China; E-Mail: awag7674@fimmu.com; 4 Department of Dematology, Daping Hospital, Third Military Medical University, Chongqing 400042, China; E-Mail: pharsunr@126.com

**Keywords:** lung-targeted, emodin, microspheres, polylactic acid, sustained-release

## Abstract

Emodin (1,3,8-trihydroxy-6-methylanthraquinone) has been identified to have the potential to improve lung fibrosis and lung cancer. To avoid the liver and kidney toxicities and the fast metabolism of emodin, emodin-loaded polylactic acid microspheres (ED-PLA-MS) were prepared and their characteristics were studied. ED-PLA-MS were prepared by the organic phase dispersion-solvent diffusion method. By applying an orthogonal design, our results indicated that the optimal formulation was 12 mg/mL PLA, 0.5% gelatin, and an organic phase:glycerol ratio of 1:20. Using the optimal experimental conditions, the drug loading and encapsulation efficiencies were (19.0 ± 1.8)% and (62.2 ± 2.6)%, respectively. The average particle size was 9.7 ± 0.7 μm. *In vitro* studies indicated that the ED-PLA-MS demonstrated a well-sustained release efficacy. The microspheres delivered emodin, primarily to the lungs of mice, upon intravenous injection. It was also detected by microscopy that partial lung inflammation was observed in lung tissues and no pathological changes were found in other tissues of the ED-PLA-MS-treated animals. These results suggested that ED-PLA-MS are of potential value in treating lung diseases in animals.

## 1. Introduction

**Figure 1.**
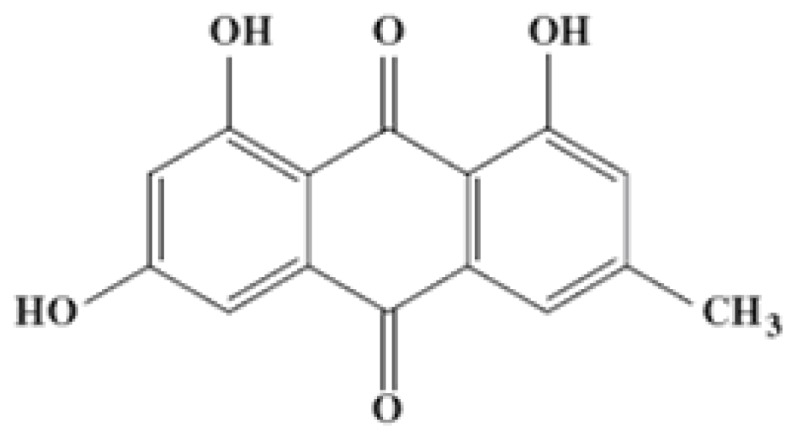
The chemical structure of emodin.

Emodin (1,3,8-trihydroxy-6-methylanthraquinone), a component of Chinese herbs, such as *Rheum officinal*e and *Polygonum cuspidatum*, is widely used in Chinese medicine [1]. The chemical structure of emodin is shown in Figure 1. It was reported that emodin can inhibit immune responses and improve hepatic fibrosis [1,2]. With regarding to lung diseases therapy, it was identified that this agent has a protective effect against the development of bleomycin-induced lung fibrosis in mice [3], and may be a potential medicine against lung cancer [4]. For the treatment of these lung disorders, the desired therapeutic agent must be administered over an extended duration. However, it has been reported that the free forms of emodin were predominant in kidney and liver [5]. Moreover, long-term use of emodin may generate many unwanted side effects, such as renal tubule adenoma, renal failure, and liver cancer [6,7]. In addition, previous studies indicated that after systemic delivery, the *T*_1/2α_ and *T*_1/2β_ of emodin are only 5.3 ± 2.9 min and 37.4 ± 6.7 min [8], respectively. To avoid the unwanted off-target effects and prevent rapid clearance of the agent, a targeted and sustained release drug delivery system is a feasible tactic.

Lung-targeted drug delivery systems can deliver drug to the lung via pulmonary inhalation or intravenous administration. Pulmonary inhalation has been receiving great attention in recent years. However, drugs could hardly reach disease locations in the periphery lungs via inhalation due to airways obstructing by inflammation or mucus plugs. Furthermore, there has been administrated acute toxicity of nanoparticles to the epithelia via the pulmonary route of delivery system. Therefore, intravenous administration of lung-targeted drug has become a more interesting field in recent years [9].

In the present work, we encapsulated emodin in microspheres using the carrier polylactic acid (PLA), a biodegradable polymer. The emodin-loaded PLA microspheres (ED-PLA-MS) demonstrated sustained release efficacy *in vitro* and we found that the microspheres accumulated mainly in the lung after intravenous injection.

## 2. Results and Discussion

### 2.1. The Characteristics of ED-PLA-MS

Various strategies, such as polymeric nanoparticles, microspheres, liposomes, and solid lipid nanoparticles, have been investigated for the sustained delivery of therapeutic agents to the lungs. Among these methods, biodegradable microspheres have been widely used for decades [10,11].

PLA is a biocompatible and biodegradable polymer with demonstrated safety that is used in a variety of Food and Drug Administration (FDA)-approved therapeutic devices. PLA is hydrolytically degraded into non-toxic oligomers or monomers of lactic acid after absorption, and has been extensively studied for the development of lung-targeted microspheres [12]. Thus, it was used as the drug carrier in our study.

The microspheres had different surface characteristics and drug loading efficiencies based on the experimental conditions. To identify the optimal formulation and process parameters for the preparation of microspheres, we first investigated the influence of the drug:PLA ratio on the microsphere preparation. The results of the drug loading and encapsulation efficiencies indicated that 1:3 was the optimal ratio.

We next used an orthogonal design to optimize the concentrations of PLA and gelatin, as well as the ratio of organic phase:glycerol. As shown in Table 1, the results indicated that the optimal formulation was 12 mg/mL PLA, 0.5% gelatin, and an organic phase:glycerol ratio of 1:20. Microspheres prepared using the optimal experimental conditions were globular in appearance and dispersed well. And the average drug loading and the average encapsulation efficiency were (19.0 ± 1.8)% and (62.2 ± 2.6)%, respectively.

### 2.2. Analysis of Appearance and Size Distribution of ED-PLA-MS

It is demonstrated that the *in vivo* deposition of microspheres depends largely on the particle size. Following intravenous administration, microspheres between 5 and 20 μm are trapped in the capillary bed of the lungs by the simple process of mechanical entrapment, thereby achieving passive lung-targeting [13].

**Figure 2.**
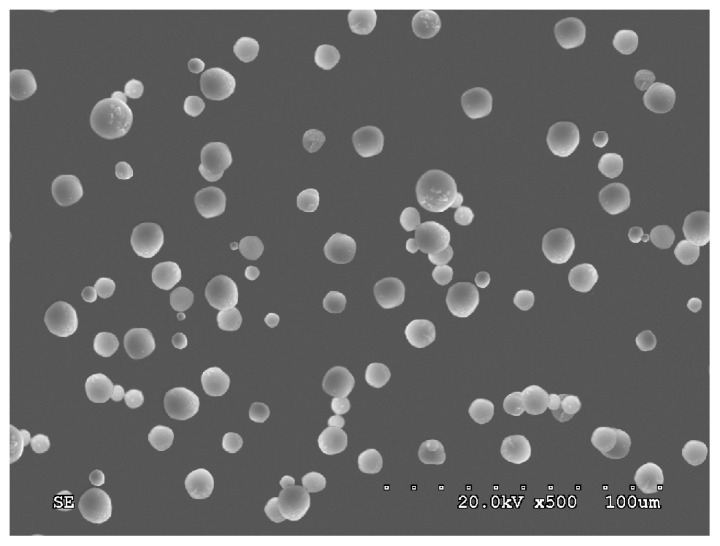
Emodin-loaded microspheres observed by scanning electron microscopy. The particles were prepared by affixing double-sided carbon tape to SEM mounts. Adhered particles were imaged after gold-sputtering. The scale bar represents 100 μm. SE = scanning electronmicroscopy.

In our study, ED-PLA-MS prepared by the organic phase dispersion-solvent diffusion method were discrete, spherical, and had a smooth surface as observed by scanning electron microscopy (Figure 2). In this study, the average particle size was 9.7 ± 0.7 μm, and 86% of the microspheres were within the size range of 5–20 μm. The results indicated that the microspheres will primarily accumulate in the lung after intravenous injection.

### 2.3. The Stability of Emodin Microspheres

During storage at 3–5 °C or at room temperature (15–25 °C) for 6 months, the surface morphology and drug contents showed no notable changes (see Table 2).

### 2.4. In Vitro Release Characteristics of ED-PLA-MS

**Figure 3.**
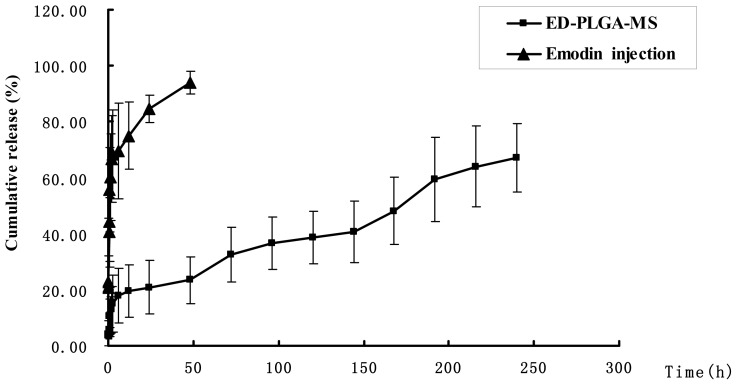
Cumulative amount of emodin release from the ED-PLA-MS in PBS (pH 7.4) containing 10% alcohol. *In vitro* release kinetics was carried out at 37 ± 1 °C by the dialysis bag technique. Emodin release from stock solution was studied as control. Data as mean ± S.D., *n* = 3.

Figure 3 shows the emodin release curve from the ED-PLA-MS and emodin solution. In comparison with emodin solution, emodin release from microspheres was biphasic (Figure 3), consisting of a burst phase (up to 12 h), sustained release (12–192 h). Of the total emodin in the ED-PLA-MS, 23.46% was released in the first 12 h, which may reflect the significant amount of emodin adsorbed on, or incorporated near, the surface of the microspheres. In clinical practice, it will bring about fast effects to the patients. After the fast release of the first 12 h, emodin released from PLA microspheres was very slow. After five days (120 h), 48.19% of emodin was released from microspheres, whereas only 67.06% release was observed after 8 days (192 h). In contrast, the release of emodin from the emodin solution was very fast. More than 55% of emodin was released at 1 h. After 48 h, the cumulative release rate approximately reached 94% (Figure 3). The data obtained from *in vitro* release studies fitting to the Higuchi model showed a good correlation (Figure 3, *r* = 0.9795) [14]. Collectively our results indicate that ED-PLA-MS had a well-controlled release efficacy.

Emodin is practically insoluble in water and soluble in alcohol [15], like peimine, the component extracted from fritillaria, a plant for the therapy of cough in China. According to Jiang [16], in the release study of the peimine gelatin microspheres, 40% ethanol is used in the release media to help enhancing diffusion of peimine successfully. In our preliminary experiment, we used PBS as the release media. However, emodin could not be detected in the release media from 0.16 to 192 h. Thus, we had to increase the ratio of ethanol in the release media from 2%, 5%, to 10%. The results indicated that emodin could only be detected when 10% ethanol was used. Thus, 10% ethanol is used in the release media in our study.

### 2.5. In Vivo Emodin Distribution Studies

**Figure 4.**
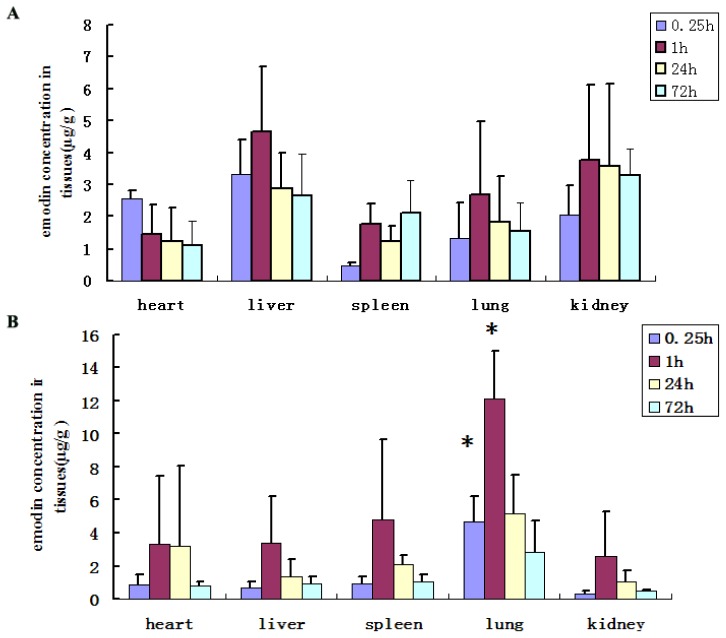
Concentration of emodin in tissues at different time following tail intravenous administration of (**A**) emodin solution or (**B**) ED-PLA-MS in mice. * *p* < 0.01 *vs.* emodin solution group.

Emodin is widely used in China in clinical practice. However, it has been reported that long-term use of emodin may result in severe diarrhea and liver or kidney cancer, which may lead to acute renal failure [6,7]. To avoid the possible toxic effects on other organs, targeting emodin to lung tissues was an optimal approach. Based on *in vitro* observations, the size of the microspheres was in the range required for lung targeting. Thus, we administered the ED-PLA-MS or emodin solution to mice intravenously. The drug concentration in various tissues was determined by UV spectrometry. The results indicated that in the emodin solution group, the concentration of emodin in the liver and kidney tissues was higher than in other tissues (Figure 4A). In contrast, the ED-PLA-MS delivered emodin primarily to the lung after intravenous injection, and the concentration of emodin in the lung was significantly higher than these in other tissues at 1 h (*p* < 0.01, Figure 4B).

Based on the above observations, we successfully prepared ED-PLA-MS by the organic phase dispersion-solvent diffusion method, which showed both proper lung-targeted and sustained drug release characteristics.

There are many reports that focus on the preparation of lung-targeted microspheres loaded with antibiotics, anticancer drugs, or glucocorticoids for the successful treatment of tuberculosis, tumors, pneumonia, and lung injury [17–20]. However, few studies reported the preparation of lung targeting and sustained drug delivery microspheres for the treatment of lung fibrosis.

Our studies showed that no pathological changes were found in the heart, liver, spleen, or kidney tissues of the ED-PLA-MS-treated animals as observed by microscopy, which indicated that the lung-targeted microspheres may avoid the off-target toxicities previously reported for emodin. Meanwhile, partial inflammation was observed in the lung tissues after injection of the emodin-loaded microspheres formulation. The inflammatory cells consisted of lymphocytes, microphages and neutrophils. As the inflammation also appeared in the lung tissues of mice treated with blank PLA microspheres, it may be due to the embolism of a number of blood vessels by the microspheres at the same time [9]. On the other hand, no cell morphology changes were observed after the microspheres injection. In addition, the organ weights did not change significantly.

After intravenous administration in mice, the microspheres were gradually swelled and the drug was released. Recently, Chao *et al.* systematically studied the relationship of rigid microsphere size with lung targeting efficiency, intralung distribution, and retention time. They indicated that complete entrapment and retention of 10 μm microsphere was observed for 1-week duration [21]. On the other side, our *in vitro* results of ED-PLA-MS showed sustained release over eight days. Therefore, the ED-PLA-MS prepared in this study may be administrated to mice once more than one week.

## 3. Experimental Section

### 3.1. Chemicals and Reagents

The following materials were used: emodin (purity > 98% by high-performance liquid chromatography, Xian Acetar Biotech Pharmaceutical Company, Xi’an, China), and PLA (*M_W_* = 20,000, Shenzhen Guanghua Weiye Industrial Co., Ltd., Shenzhen, China). All other regents were of analytical grade.

### 3.2. Microsphere Preparation

ED-PLA-MS were prepared by the organic phase dispersion-solvent diffusion method as previously described by Zhang *et al.* [22] with slight modifications. Briefly, according to the formulas listed in Table 1, 20 mg emodin and 60 mg PLA were dissolved in a 5 mL:2.5 mL mixture of dichloromethane–acetone, which constituted the organic phase, by stirring at 500 rpm. A volume of 150 mL of glycerol was added to the solution and stirred for 10 min to form an evenly distributed suspension. Then, the mixture was poured into 80 mL 0.5% (*w*/*v*) gelatin and homogenized at a rate of 500 rpm for 10 min to remove the organic solvent. After incubation at room temperature for 30 min, ED-PLA-MS were collected by centrifugation at 3000 rpm for 15 min, washed four times with distilled water, and lyophilized for 24 h under reduced pressure with phosphorus pentoxide as desiccant. PLA microspheres (PLA-MS) without the addition of emodin were also prepared as a control using the same procedure.

The concentration of PLA and gelatin and the ratio of organic phase to glycerol were investigated for their effects on emodin loading and entrapment. Orthogonal design methods for selecting the optimal formula and process parameters were applied.

### 3.3. Morphology and Size Distribution Analysis

The microsphere morphology was observed by optical microscopy. In addition, high-resolution images were obtained of the lyophilized particles using a Hitachi S2510 scanning electron microscopy (SEM, Tokyo, Japan). Samples were prepared by affixing double-sided carbon tape to SEM mounts. Adhered particles were imaged after gold-sputtering. Five hundred particles were measured for particle size distribution and measurements were carried out using optical microscopy [22].

### 3.4. Emodin Loading and Encapsulation Efficiencies

The emodin loading was determined as previously described [19,22]. Briefly, emodin was extracted from the microspheres with dichloromethane, then, the emodin concentration was determined at 295 nm using a Shimadzu UV21601 spectrophotometer with an emodin calibration curve (1.04–52 μg/mL concentration range in dichloromethane). The calibration curve was *A* = 0.058*C* − 0.0057, *r* = 1. The method recovery was (99.99 ± 0.23)%.

Emodin loading was expressed as mg drug/100 mg of ED-PLA-MS (%, *w*/*w*). The experiments were conducted in triplicate.

The emodin entrapment efficiency (%) was calculated as following: Emodin loading ratio × the quantity of emodin-loaded microspheres/the emodin quantity added in the microsphere preparation process.

### 3.5. The Stability of ED-PLA-MS

The microsphere powders were kept for 6 months at 3–5 and 15–25 °C, respectively. The microsphere morphology and drug content were tested periodically.

### 3.6. In Vitro Drug Release

The *in vitro* drug release from microspheres was determined in 0.01 mmol/L phosphate-buffered saline (PBS, pH 7.4) containing 10% alcohol according to Jiang [16]. One milliliter of emodin-loaded microspheres was filled into a dialysis tube (molecular weight cut-off = 12,000), and the end sealed dialysis tube was immersed fully in 50 mL of the release medium. The ED-PLA-MS dispersion was incubated at 37 ± 1 °C under magnetic stirring at 50 rpm. At predetermined time points, 1 mL of the release medium was withdrawn and replaced with an equal volume of fresh release medium [14]. The emodin contents were determined spectrophotometrically at 295 nm, and the concentrations were calculated using a calibration curve (*A* = 2.6063 − 0.0259*C*, *r* = 0.9971) prepared in PBS containing 10% alcohol. All release experiments were performed as triplicates. The results of all measurements were used to calculate cumulative drug release. The release behavior of emodin from microspheres was analyzed with the Higuchi model.

### 3.7. Tissue Distribution of Microspheres

Pathogen-free C57 Bl/6J mice of either sex, weighing between 20 and 22 g were obtained from the Laboratory Animal Center of Sun Yatsen University (Guangzhou, China). All animals were maintained in a specific pathogen-free environment at 23 ± 2 °C temperature with free access to water.

All animals were divided into two groups. The control group was treated with emodin solution (dissolved in physiological saline containing 0.5% DMSO), and the other group was administered with ED-PLA-MS suspension (dispersed in physiological saline). Each formulation was given intravenously at a dose of 10 mg/kg emodin. Mice were sacrificed at 15 min, 1 h, 24 h, and 72 h after administration. When the mice were sacrificed, the tissues of interest (heart, liver, spleen, lung, and kidney) were collected after being washed using normal saline and dried with tissue paper to remove excess fluid.

The isolated tissues were weighed accurately and homogenized in cold 0.01 mmol/L PBS buffer (pH 6.8, weight:volume = 0.2 g:1 mL). Following centrifugation of the homogenate, the emodin in the supernatant was extracted with 2 volumes of dichloromethane, and the emodin concentration was determined by UV spectrometry as mentioned above. The calibration curves used were as follows: *A* = 2.2700*C* − 0.0030, *r* = 0.9993 (for heart tissue); *A* = 2.0096*C* − 0.0031, *r* = 0.9993 (for liver tissue); *A* = 2.2700*C* − 0.0030, *r* = 0.9912 (for spleen tissue); *A =* 2.1317*C* − 0.0017, *r* = 0.9940 (for lung tissue); *A* = 2.1317*C* − 0.0017, *r* = 0.9897 (for kidney tissue).

### 3.8. Histopathological Studies

Seventy-two hours after intravenous administration of ED-PLA-MS to C57 Bl/6J mice at a dose of 10 mg/kg emodin, the organs, including heart, liver, spleen, lung, and kidney were collected. Then fixed by inflation with 4% paraformaldehyde in PBS (pH 7.4) for 24 h and embedded in paraffin, finally H&E hematoxylin and eosin staining were performed. The pathological changes were detected by microscopy in all these tissues.

### 3.9. Statistical Analysis

Data were expressed as means ± standard deviation (S.D.). Statistical analyses were carried out using an ANOVA followed by appropriate *post hoc* tests, including a multiple comparison tests (least significant difference). All analyses were performed using SPSS version 12.0 software package (Chicago, IL, USA). Differences were considered to be statistically significant at *p* < 0.05.

## 4. Conclusions

In this study, emodin-loaded sustained-release PLA microspheres were developed. The microspheres showed a combination of lung-targeted and sustained drug release characteristics. In addition, it may avoid the off-target toxicities previously reported for emodin. Our study has, thus, demonstrated that emodin-loaded sustained-release PLA microspheres are of potential value in treating lung fibrosis in animals.

## Figures and Tables

**Table 1.** Preparation and characterization of polylactic acid (PLA) microspheres prepared from different formulas using the solvent emulsion-evaporation method (mean ± SD, *n* = 3).

Formula	PLA (mg/mL)	Organic phase: glycerol	Gelatin (%, *w*/*v*)	Encapsulation efficiency (%)	ED loading (mg/100 mg)	Size (μm)
MS1	8	1:10	0.1	54.2 ± 0.3	14.2 ± 0.3	11.3 ± 0.7
MS2	8	1:20	0.5	59.1 ± 0.3	15.3 ± 0.3	9.3 ± 0.6
MS3	8	1:30	1.0	52.4 ± 2.0	16.2 ± 1.2	8.2 ± 0.3
MS4	12	1:10	0.5	63.7 ± 3.1	16.8 ± 1.7	11.5 ± 1.2
MS5	12	1:20	1.0	62.3 ± 2.8	16.5 ± 2.1	10.0 ± 2.0
MS6	12	1:30	0.1	61.2 ± 2.5	15.6 ± 1.2	12.1 ± 2.1
MS7	16	1:10	1.0	53.9 ± 2.1	16.2 ± 2.1	11.1 ± 1.6
MS8	16	1:20	0.1	58.2 ± 0.3	15.3 ± 0.3	12.6 ± 0.6
MS9	16	1:30	0.5	56.5 ± 0.3	16.2 ± 0.3	11.3 ± 0.4

**Table 2.** Content of emodin-loaded polylactic acid microspheres (ED-PLA-MS) in various storage conditions (*n* = 3).

Temperature (°C)	Time (month)	Drug loading ± S.D. (%)
3–5 °C	0	19.2 ± 1.7
	2	19.2 ± 1.6
	4	19.3 ± 0.8
	6	19.2 ± 2.0

15–25 °C	0	19.2 ± 1.7
	2	19.3 ± 0.9
	4	19.2 ± 1.6
	6	19.1 ± 1.6
